# Codesigning simulations and analyzing the process to ascertain principles of authentic and meaningful research engagement in childhood disability research

**DOI:** 10.1186/s40900-022-00398-y

**Published:** 2022-11-09

**Authors:** Samantha K. Micsinszki, Nadia L. Tanel, Julia Kowal, Gillian King, Dolly Menna-Dack, Angel Chu, Michelle Phoenix

**Affiliations:** 1 School of Rehabilitation Science, McMaster University, Hamilton, ON Canada; 2 CanChild Centre for Childhood Disability Research, Hamilton, ON Canada; 3 Bloorview Research Institute, Toronto, ON Canada; 4 University of Toronto, Toronto, ON Canada

**Keywords:** Simulation, Patient engagement, Family engagement, Patient-oriented research, Childhood disability, Authentic, Meaningful

## Abstract

**Background:**

Including youth with disabilities and their families as partners in childhood disability research is imperative but can be challenging to do in an authentic and meaningful way. Simulation allows individuals to learn in a controlled environment and provides an opportunity to try new approaches. The objectives of the research study were to (1) codesign a suite of simulations and facilitation resources and understand how stakeholders engaged in the codesign process; and (2) describe the principles of authentic and meaningful research engagement as identified by stakeholders.

**Methods:**

Interdisciplinary stakeholder groups, including youth with disabilities, parents, researchers, and trainees, codesigned simulation training videos by developing shared storylines about challenges with research engagement that were then performed and digitally recorded with standardized patient actors. Two forms of data were collected: (1) observations via field notes and video recordings were analyzed to understand the codesign process; and (2) interviews were analyzed to understand principles of authentic and meaningful engagement.

**Results:**

Four simulation training videos were developed, and topics included: (1) forming a project team; (2) identifying project objectives and priorities; (3) reviewing results; and (4) navigating concerns about knowledge translation. Thirteen participants participated in the simulation codesign; nine of whom consented to be observed in the codesign process and seven who completed follow up interviews. We identified two themes about authentic and meaningful engagement in research: (1) whether the invitation to engage on a project was authentic and meaningful or was extended to ‘tick a box’; and (2) whether there were authentic and meaningful opportunities to contribute (e.g., valued contributions aligned with people’s lived experience, skills, and interests) or if they only served as a ‘rubber stamp’. Communication and expectations tied the ‘tick box’ and ‘rubber stamp’ themes together and underlie whether engagement was authentic and meaningful.

**Conclusions:**

For research engagement to be authentic and meaningful, researchers and families need to set clear expectations, build rapport, have tangible supports, use clear communication, and build time and space to work together. Future work will explore the utility of the simulations and whether they improve knowledge and attitudes about authentic and meaningful engagement in research.

**Supplementary Information:**

The online version contains supplementary material available at 10.1186/s40900-022-00398-y.

## Plain English summary

Researchers, patients, and families who collaborate in childhood disability research can benefit from training on how to engage with each other authentically and meaningfully, i.e., where all parties feel supported and valued. We used a codesign approach to identify aspects of the research process where challenges might arise between researchers, patients, and families and then developed four videos with scenarios that mimic these challenges. Codesign is a collaborative approach in which different perspectives and relationships are prioritized while working to achieve a common aim. First, researchers, youth with disabilities, families, and trainees each identified challenges they had previously experienced in research engagement and used those to create one common scenario as the premise of each video. In follow up interviews, we asked a subset (7 people) of those who took part (13 people) about their experience in the co-design process and about what it means to engage in research where all parties feel supported and valued. Participants said that being invited to partner on research teams needed to be more than just a ‘tick box’ and even when invited onto research teams, they often lacked ways to contribute in a way where they felt valued. Engagement felt like a ‘rubber stamp’ when they were asked to contribute in a narrow way that did not align with the fullness of their lived experience, skills, and interests. Clear communication and mutual expectations were important for engagement to happen in a way that felt supportive and valuable. We suggest that researchers and families need to set clear expectations, build rapport, have tangible supports, use clear communication, and build time and space to work together.

**Supplementary Information:**

The online version contains supplementary material available at 10.1186/s40900-022-00398-y.

## Background

Involving patients and their families in the design, conduct, and dissemination of research is a central component of patient engagement, patient-oriented research (POR), and codesign. Despite the various terms used to describe the engagement of people with lived experience in research [1], this article uses the term POR, which refers “to a continuum of research that engages patients as partners, focusses on patient-identified priorities and improves patient outcomes…[and] aims to apply the knowledge generated to improve healthcare systems and practices” [2, p. 5]. Including people with disabilities as ‘patient partners’ (i.e., people with lived experience partnering on a research team) and their caregivers, or other family members as ‘family partners’ [2] advances research that is relevant and focused on the priorities of the communities being represented [3, 4]. Including patients and families in the research process has potential benefits to the research project, such as suggestions for interventions that may lead to better health outcomes [2], and improved study enrollment and decreased participant withdrawal [5, 6]. POR also has positive personal benefits for patient partners [7], such as improved research skills [3], internal validation of their lived experiences [8], and increased confidence and self-esteem [9].

Although there is increasing evidence to support the benefits of engaging with people with lived experience throughout the research process, recent research has identified that researchers and patient partners often do not have all the required skills (e.g., communication, interpersonal etc.) and training to form meaningful research partnerships [8, 10]. A lack of understanding and awareness of how to engage meaningfully can lead to tokenistic, inauthentic, and failed research partnerships [8, 11–13]. Meaningful engagement is defined by Hamilton et al. [14] as “the planned, supported and valued involvement of patients in the research process…that facilitates effective contributions by patients or their surrogates to help to produce important outcomes while benefitting the patients” (p. 404). For engagement to be meaningful and authentic, it should be inclusive, transparent, purposeful, and value multiple kinds of knowledge and experience [15]. Strategies to engage a variety of perspectives should be used (e.g., offering different methods of engagement) [16] and stakeholders should be engaged throughout the research process where the resulting partnerships should be “deep, extensive and long-lasting” (p. 294) [17]. However, meaningful engagement is not easy [18] and requires additional time, thought, and action, particularly from researchers, to overcome potential challenges of involving patient partners on research teams [5, 13]. For example, there is a need to set clear and realistic expectations for all parties [8]; ensure that patient partners feel comfortable and prepared to provide meaningful input [8]; elicit meaningful input [10]; and address opposing views or opinions.

To engage in meaningful research, core values of patient engagement and the codesign process, such as trust and reciprocity, and skills in communication are needed from researchers, patients, and families [19–21] to promote safe and inclusive research spaces. These values and skills are not typically included in researcher training programs and the lack of competency in these areas is often described as a barrier to engaging with patient partners by researchers interested in POR [22–24]. There are stand-alone training programs for engaging patients as partners in research, such as the Patient Oriented Research Curriculum in Child Health (PORCCH) [25], the Kids Brain Health Network, CanChild, McMaster’s Continuing Education Family Engagement in Research (FER) Course [26], Partners in Research (PiR) [27], and the Patient and Community Engagement Research (PaCER) [28] training program. While programs tend to take the form of online modules or certificate courses, which are helpful resources to teach the basic principles of research and the concept of POR, few have been designed for patient partners and researchers to learn together in a safe environment where they experience firsthand each other’s challenges. Codesign is a dynamic and inclusive approach that incorporates a variety of experiences and perspectives, such as patients and families, as well as researchers and service providers. As a relational process [29], codesign values all knowledge and perspectives, builds connections and trust across different stakeholders involved in the process, and requires ongoing collaboration [30]. In this article, the term codesign is used to represent the centering of patient and family perspectives where participant experiences are elicited through storytelling to help design solutions to existing problems [31, 32].

To optimize engagement and ensure successful partnerships, the literature suggests that education and training are needed for patient partners and researchers *together* in an environment that supports trust, respect, reciprocity and co-learning [10]. Simulation is a tool that allows for this type of learning environment, particularly within the context of codesign. Zubairi et al. [33] proposed the use of simulation as a tool that allows learners to recreate challenging situations and learn from these experiences in a controlled environment. Simulation as an educational tool uses “devices, trained persons [i.e., actors, Standardized Patients (SPs)], lifelike virtual environments, and contrived social situations to mimic problems, events or conditions that arise in professional encounters” [31, p. 11]. During and directly after a simulation, learners are guided through rich discussions and can reflect on the scenario, rethink and reshape their learning [34]. Through simulation, with appropriate facilitation and debriefing, individuals learn in an environment where trust and respect are established and groups are encouraged to experience vulnerability and humility [35, 36].

Although some existing courses and curricula support learning the principles of POR with both researchers and patient and/or family partners, we are not aware of any that incorporate simulation. Simulation training may expand existing POR training opportunities because it is well suited to (1) address the relationship-based nature of the challenges that can exist in POR; (2) explore and empathize with diverse perspectives; (3) practice responding in real-time; and (4) build in opportunities for critical reflection and feedback. Simulations’ learning objectives depend largely upon who is involved in the building process. Engaging relevant patient and family partners in the codesign of simulations, an approach in which patient partner perspectives are included throughout the process [37, 38], is seen as an important way to “intentionally model the environment and interactions that a POR team would likely be facing in the ‘real world’” [36, p. 4].

Overall, the purpose of this work was to develop a suite of simulations and accompanying facilitation resources to advance authentic and meaningful engagement in childhood disability research between researchers and patient and family partners. The objectives of the research study were to (1) codesign a suite of simulations and facilitation resources and understand how stakeholders engaged in the codesign process; and (2) describe the principles of authentic and meaningful research partnerships as identified by stakeholders in childhood disability research.

## Methods

### Ethics approval

Institutional ethics approval (REB#19-859) was received.

### Study design

This study used an intrinsic case study methodology [39, 40] to describe how a diverse group of people codesigned simulations and constructed principles of authentic and meaningful engagement in childhood disability research partnerships. Multiple data sources were used to observe the codesign process and interview participants about their experiences with authentic and meaningful engagement. This initiative was reported according to the Guidance for Reporting Involvement of Patients and the Public (GRIPP) 2.0 [41] (see Additional file 1).

### Sample and recruitment

This case study was bound by place and activity [39]. The case study was situated at a large pediatric rehabilitation hospital in Canada that has a culture and history of supporting patient and family engagement in the research process. Purposeful sampling [42] was completed at two Canadian disability research centres to recruit patient and family partners who had partnered with research teams in non-participant roles or capacities (e.g., advisor, collaborator etc.) in the last five years. This group included individuals 16–29 years of age who had a childhood onset disability or acquired injury (e.g., autism, cerebral palsy, acquired brain injury, physical disability, complex needs etc.), consistent with the definition of youth at the pediatric hospital, or their parents or primary caregivers. We also recruited researchers (e.g., Ph.D.-trained scientists, postdoctoral fellows), trainees (e.g., Master’s and Ph.D. thesis-based students), and research staff (e.g., research assistants, research coordinators etc.) whose primary research focus was on childhood disability and who had worked with youth with disabilities and/or parents/primary caregivers as patient or family partners, respectively. All participants were recruited using organizational email lists, newsletters, and/or social media. Individuals were provided with an option to participate in the simulation codesign process with or without the research component. Those who did not consent to participate in the research were put into a group together (n = 4), while participants who consented to participate in the research (n = 9) were divided into two roughly equal groups. All groups had representation from five different stakeholder categories (i.e., researchers; research staff; trainees; parents of children with disabilities; and youth with disabilities).

### Simulation codesign process

#### Creation of initial prompt scenarios

The research team, which was comprised of two parents with experience in POR, interdisciplinary researchers, and a simulation/education expert, initially developed five scenarios to serve as prompts for the codesign sessions. To develop the prompt scenarios, the team reflected on their experiences partnering in childhood disability research. Similar to other simulation studies [33, 36], the research team specifically focused on the challenges that can occur during an interaction. The research team then sequentially organized these challenges according to the traditional research process (e.g., question identification through to dissemination), with hopes that the simulations could be used by project teams regardless of which phase of a research project they were partnering in. Each research team member added details about specific challenges they had experienced on a ‘research process map’ and commented on the entries of other team members. All team members attended a meeting to reflect on how the challenges aligned with the literature, to synthesize the challenges, and to select the priorities for further exploration and development through the simulation codesign process.

#### The simulation codesign event

One week prior to the simulation event, initial prompt scenarios were pre-circulated to participants to stimulate reflection. These scenarios were provided to groups to guide discussion, and to prevent topic overlap between groups (i.e., to ensure four different videos were created), but were not meant to restrict the simulation codesign. A one-day simulation event that included a three-part simulation codesign process occurred in January 2020 with one to two individuals from each of the five stakeholder categories (i.e., researchers; research staff; trainees; parents of children with disabilities; and youth with disabilities). At the start of the simulation event, a group orientation was provided that included an overview of the project background and purpose as well as an introduction to simulation and its use in capacity building. Then the simulation codesign process happened; part one occurred during the morning of the event and parts two and three occurred in the afternoon, as described below. Lunch was provided between the morning and afternoon sessions, and renumeration for transportation (i.e., train or bus ticket, parking) was available. Participants were offered a gift card honorarium.

#### Part one of the simulation codesign

Three groups of four to five participants were formed, and given one to two of the pre-circulated scenarios representing challenging situations in POR. These scenarios were intentionally broad (e.g., ‘during the planning phase of a research study, clients, families, and researchers may disagree about the research question or objectives’) (see Additional file 2); this provided space for a variety of experiences and perspectives to be discussed. Individuals were then instructed to share a story relevant to the scenario(s) from their own experience with their group. Each group was provided a handout of five guiding principles of family engagement (i.e., inclusiveness, co-development, transparency, support, and mutual respect) developed at Holland Bloorview Kids Rehabilitation Hospital [43]. The principles were to be used as a framework to ground the developing simulations. After all stories were shared, each group worked collaboratively to create a short, common storyline that was reflective of stakeholders’ experiences in that scenario [44, 45]. During this time, a simulation facilitator moved between the groups to provide support. All groups were given two hours to complete part one of the simulation codesign.

#### Part two of the simulation codesign

Standardized patient actors (i.e., professional actors trained in simulation methodology) and a simulation facilitator then joined each group to provide nuance to the storylines developed in part one. The group members and standardized patients collaborated to (1) confirm the acted storylines portrayed the identified challenges; (2) ensure the authenticity of the simulations; and (3) provide an opportunity for the creators to tweak the simulations into a final product for recording. This included asking key questions so that the standardized patients could understand the background and motivations of the characters they were portraying in the scenario.

#### Part three of the simulation codesign

**Fig. 1:**
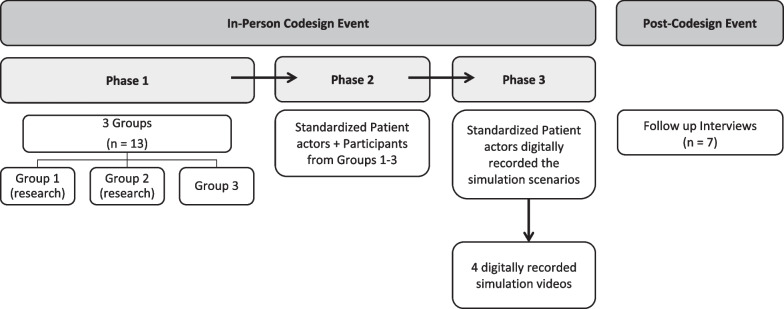
Diagrammatic representation of the phases of the codesign project

The four simulations were acted out by the standardized patient actors with each group having at least two actors playing different roles (e.g., researcher, family member, etc.). The simulations were then digitally recorded. A few months after the event, the research team met as a group several times to review the final simulation recordings and discuss key themes from each of the simulations. A facilitator guidebook was developed by the research team with instructions on how to facilitate each simulation. An overview of the codesign process is depicted in Fig. 1.

### Patient engagement in this research study

All authors worked collaboratively throughout this research study. Two parents of children with disabilities who were previously known to the research team were engaged as partners throughout the research process. This included providing input and feedback on the development of the research question, supporting participant recruitment and data collection, and providing written feedback on study documents and the written manuscript. A youth partner joined the research team after data collection was complete and provided feedback on data analysis and supported manuscript writing. While some engagement activities occurred in-person (e.g., the simulation event) patient and family engagement as part of our research team occurred primarily through email and online discussions via phone or Zoom. Even prior to the COVID-19 pandemic, partners were provided the opportunity to join project meetings by phone or Zoom and provide feedback in a format they preferred (e.g., through discussion, via email, or tracked changes). Outcomes, contexts, and processes of patient engagement were not formally captured or evaluated during this research study.

### Data collection

We collected two forms of data: (1) observations via field notes (in person) and video recordings (objective 1) and (2) interviews (objective 2). Demographic information was collected from all research participants in part one of the simulation codesign via a written questionnaire.

#### Objective 1

Two of the three simulation codesign groups were video recorded (audio and video) during the in-person story-sharing exercise in part one of the simulation codesign. Two members of the research team with qualitative research experience (SM and MP) used a field note template to take observational field notes of the two groups participating in the research during phase one of the simulation codesign. Observations were gathered to describe the setting, participants, activities, and interactions [39]. Video recordings were used to capture what was said among participants and the researcher team as part of the simulation codesign and what was ‘not said’ but ‘done’ (e.g., interactions, movements, facial expressions, etc.) [46, 47]. Additionally, videos were used to understand the intersubjective and/or social aspects of simulation codesign collaborative-research [48].

#### Objective 2

Virtual semi-structured interviews were completed approximately two months after the codesign event (a delay due to the onset of COVID-19). Interviews were conducted by one member of the research team with qualitative interview experience (SM) with a subset of participants who agreed to be contacted to take part in an interview. In the interviews, we asked participants to reflect on their previous experiences engaging in research and the experience of taking part in the simulation codesign. Interview questions were generated based on the themes emerging from the analysis of the video data. The purpose for conducting the interviews was to probe for new insights into themes related to meaningful and authentic collaboration in research and to explore perspectives about participating in the codesign process.

### Data analysis

#### Objective 1

The observers first compared their field notes and wrote a summary to comprehensively describe the context and situate the case. To determine how stakeholders were engaged in the codesign process, two members of the research team (SM, MP) descriptively analyzed the videos, observations, and the interview data that pertained to the codesign process. A coding document was used when analyzing the videos of the codesign sessions to capture indicators of social interaction (e.g., social acts of commenting, interrupting, questioning etc.) [46, 47] and non-verbal communication (e.g., smiles, laughter, gestures, proximity between clients, exaggerated pauses, material sharing) [49]. Two of the co-authors (SM, MP) analyzed the video data from part one of the simulation codesign by first watching the videos together, discussing the participants’ stories about their engagement in research, and then independently coding the indicators of social interaction and non-verbal communication on an investigator developed coding chart. SM and MP met to share their coded data, discuss agreements/disagreements, and compare the coded data across videos to develop a comprehensive summary of the relational and process elements of how people engaged in the codesign of the simulations.

#### Objective 2

To identify themes of authentic and meaningful engagement in participants’ past research experiences, thematic analysis of the interview data was completed with use of memos to familiarize ourselves with the data and diagrams to support the generation of initial themes [50, 51]. Interviews were transcribed verbatim, de-identified, and imported into the qualitative and mixed methods research web application, Dedoose [52]. Transcripts were coded by SM who had experience with qualitative methods and were analyzed using the six stage process for thematic analysis described by Braun and Clark [50] to move from the generation of initial codes to a set of themes that holistically capture and explain the data. SM and MP met bi-weekly to discuss the emergent structure and fit of the data, and to elevate the codes to themes. Memos were written to record impressions and areas to further investigate through the analytic process. The development of themes included interpretation by the research team and collaboration between all team members. Analysis occurred sequentially: videos were analyzed first, followed by the interview data, and triangulated across data sets.

## Findings

### Participants

Thirteen people took part in the simulation codesign event. Nine people participated in the observation of the codesign session. Seven people participated in the interviews. Two people did not consent to the observation or interview and solely completed the codesign process. Research study participants included three researchers or research staff members who had engaged in one to 10 POR-studies each; three parents of children with disabilities and one youth with disabilities, who had each engaged in one to four POR-studies; and two trainees who had each engaged in four POR-studies. We did not collect demographic information from the four codesign event attendees who did not participate in the research study. Most participants engaged partners either before the study began (e.g., during development and design) or during study conduct (e.g., participant recruitment, data collection). Participants had been involved in diverse projects (e.g., outcome measure development, simulation work, knowledge translation) and often engaged with multiple teams. Parents and youth were often involved as an advisor on a project or a committee, working within a project team or a larger department to provide insights from their lived experience. Researchers (e.g., research coordinators and principal investigators) and trainees had typically been involved in studies or provided support through activities such as recruitment, data collection, and knowledge translation and dissemination.

### Simulation videos

Four simulation training videos were created. The videos aim to prepare youth with disabilities, parents of children with disabilities, trainees, researchers, and research staff to develop the knowledge and skills to engage in research authentically and meaningfully. The simulation videos cover four topics, which include: (1) forming a project team; (2) identifying project objectives and priorities; (3) agreeing on results; and (4) carrying out knowledge translation. Videos range from less than three minutes to over five minutes in length and include instructions for use and a description of the scenario. The accompanying facilitator guidebook includes a brief description of each simulation, learning objectives, suggested probing questions and key points or messages that could be raised during the simulation debrief, and provides a recommended structure on how to set up and run the simulations. The simulations were developed for educational purposes and can be accessed and used free of charge through the Holland Bloorview Kids Rehabilitation Hospital Simulation Hub [53].

### Objective 1: codesign the simulation and understand how stakeholders were engaged in the process.

Diverse strategies of verbal and non-verbal communication were used during the simulation codesign. Non-verbal communicative acts, such a pausing, leaning in, and making eye contact were observed as means used to invite people into the conversation. People who took on leadership roles, typically researchers, research staff, or postdoctoral fellows, also verbally invited those who had not yet contributed much to the conversation to provide their perspectives.

Frequent non-verbal indicators of encouragement and excitement were observed, including hand clapping, nodding, facial expressions, and smiling, lending to a positive and supportive environment. Hand gestures were used as a way to support or illustrate verbal questions and responses (e.g., casting a fishing net; counting “1, 2, 3”). Verbal communication was also used to support group members (e.g., “that was a great question”) and jokes were used to create a light atmosphere or mood in the room. Both groups had interruptions (e.g., a late arrival) that disrupted the flow of conversation.

When we analyzed the videos of both codesign groups, we found the researchers (i.e., research staff member, principal investigator, and postdoctoral fellow) were the most verbal. Of the total number of communicative acts recorded, the most common across both codesign groups included asking questions (n = 163/474, 34%), making comments (n = 292/474, 62%), and voicing agreement (n = 217/474, 46%). Restating, summarizing, and giving direction were done almost exclusively by the researchers. Research team members took on the role of leading or managing the group as participants were not pre-assigned materials or roles. Those who took control of the simulation codesign prompts and worksheets also tended to control the direction of the group and were looked to as natural leaders. For example, one participant who naturally led the conversation in the video analysis also pointed this out in the follow up interview: *“*one other person in my group, I think between us, we really tried to steer the conversation. So, I think between us we sort of really handed off um sort of being the group facilitator to be honest*”* (participant 6, researcher). We also found that very little voiced disagreement occurred during the simulation codesign exercise. However, in the interviews, two participants (one researcher and one family member) described that the shared story developed by the group did not align with their experience, but this was not clearly voiced in the room.

### Objective 2: describe the principles of authentic and meaningful research engagement as identified by stakeholders

Two themes related to authentic and meaningful engagement in research were identified: (1) Invitation to Authentically and Meaningfully Engage: Not Just a ‘Tick Box’ and (2) Authentic and Meaningful Engagement: Not Just a 'Rubber Stamp'. Interview participants suggested that the themes are influenced by underlying elements of communication and expectations between researchers, youth, and parents, and can influence authentic and meaningful engagement in either positive or negative ways.

#### Theme 1: invitation to authentically and meaningfully engage: not just a ‘tick box’

Participants spoke about who is invited to participate on projects as research team members, oftentimes feeling that they are being invited on projects just to fill a ‘tick box’, such that the invitations they had received to collaborate on a project were only a means for researchers to put the partners’ name on the work. One researcher admitted that they have likely done this in the past as a product of feeling rushed to include a patient partner on a grant:

“Well, I’ve probably been guilty of doing it myself to be honest. Just to be transparent…I think sometimes, you put—you rush, rush, rush to put someone’s name on, and you do genuinely want to engage, to it like—it could be months or even years after you’ve applied for something. You have to put someone’s name on it originally and then by the time you get around to actually running the project, either something’s happened to them, or something’s changed, or, you know, so—and I do think there are researchers out there who do just put people’s names on just for a tick-box…” (participant 6, researcher)

Despite researchers’ intent to engage patients and families in authentic and meaningful ways, a lack of clear expectations from the outset of the engagement (e.g., timeline, role, commitment, interest etc.) resulted in a tokenistic experience for one parent: "sometimes I would like to be more than just a figurehead” (participant 8, parent). Two researchers spoke about an ethical motivation for engaging youth and families went beyond a ‘tick box’ invitation and moved beyond tokenistic engagement: “I think it’s essential. So, sort of philosophically, it’s important to me in an ethical and moral sense to try and make sure the research is not happening in a vacuum…” (participant 5, research staff).

Who is invited to partner on research teams was also related to equity, diversity, and inclusion, and the accessibility of research engagement opportunities. Both researchers and parents mentioned barriers that limited engagement and how a lack of diversity on research teams can lead to misrepresented outcomes: "you wind up having people who can afford being here, who are not working, who are retired and it’s not allowing those other people who perhaps would have something useful to add, meaningful to add" (participant 8, parent). Parents often do not have the time during the day to engage because of work obligations. Although researchers may try to mitigate potential barriers by offering parking, gift cards, flexible options for participation, etc. these supports are not consistently made available and may further perpetuate unrepresentative partnerships: “…it’s all fine and good for us to ask people to volunteer but we may or may not be getting representative people who have the means and the wherewithal…to do that uncompensated” (participant 5, research staff). On the flip side of this, one family partner acknowledged that conducting research after typical business hours also has impacts on the researchers and clinicians who are leading the work. Balancing accessible opportunities for engagement was thus described as a “careful dance” in which the needs of both the research and the family should be considered.

#### Theme 2: authentic and meaningful engagement: not just a 'rubber stamp'

Challenges emerged when patient and family partners were not provided with opportunities to contribute in ways that recognized their skills, interests, and knowledge (e.g., education area; career experience; technical skills such data processing and graphic design; community connections) beyond lived experience: “the value of people’s participation is sometimes not recognized, or the possible scope of their contributions is not being, you know—you’re not taking advantage of the value those people could add” (participant 5, research staff). For example, a youth participant explained that they receive many engagement requests, but these are not always reflective of the types of projects they are interested in, and which intersect with their knowledge. ‘Real contributions’ as one researcher put it, are not just about putting a name or a ‘rubber stamp’ on a project. Understanding the unique skills and interests of the patient or family partner provides space for the team to engage in meaningful conversations and support contributions beyond the partners’ insights of lived experience of disability or caregiving. One researcher explained that clear and transparent communication allowed them to understand the partners’ skill and interests, which facilitated meaningful engagement:

“…that little bit of knowledge has helped us have some really useful conversations, rather than just, like validating it…or saying, “we’ve come up with this, what do you think?” and they say “fine”, rubber stamp it and then we go ahead…which is not real collaboration” (participant 6, researcher).

In this way, research engagement invitations alone did not automatically mean that partners feel like they have engaged meaningfully. For engagement to be meaningful, one researcher suggested that they needed to first get to know the partner to understand their unique experiences and expertise so that those could then line up with how the partner is asked to contribute on the project. This researcher described this process as finding the partner’s *superpower*: “how do you find kind of their superpower to tap into so they feel like they’ve engaged properly, we feel like we’ve engaged with them and it’s really authentic?” (participant 6, researcher). Moreover, there should be a ‘fit’ or an alignment between the researcher and the patient or family partner. This fit should not only encompass the time and availability needed to engage in the project, but also the patient or family partner’s interests, skills and experiences and the project needs. For example:

“I think meaningful collaboration is being able to talk about the topic, get excited about the topic, um, like make sure it really—it fits with daily realities or living with a child with a disability, you know whatever that is, um, that it’s going to provide useful information eventually for, for children with disabilities guided by the parent” (participant 6, researcher).

Conversations between research team members and research partners about how they want to be involved is critical because not all people with lived experience want to be in full partnership roles or provide insights beyond their lived experience: “…one of the misconceptions is everyone wants to be a partner…Some people just want to be listeners and I think that’s ok when you put the decision or their ownership back in their hands” (participant 7, trainee). From families’ perspectives, meaningful engagement meant benefits to themselves and their communities: “I think mostly its giving back. That’s kind of why I became a family [partner]” (participant 2, parent).

#### Communication and expectations as elements of meaningful engagement that link the themes

**Fig. 2:**
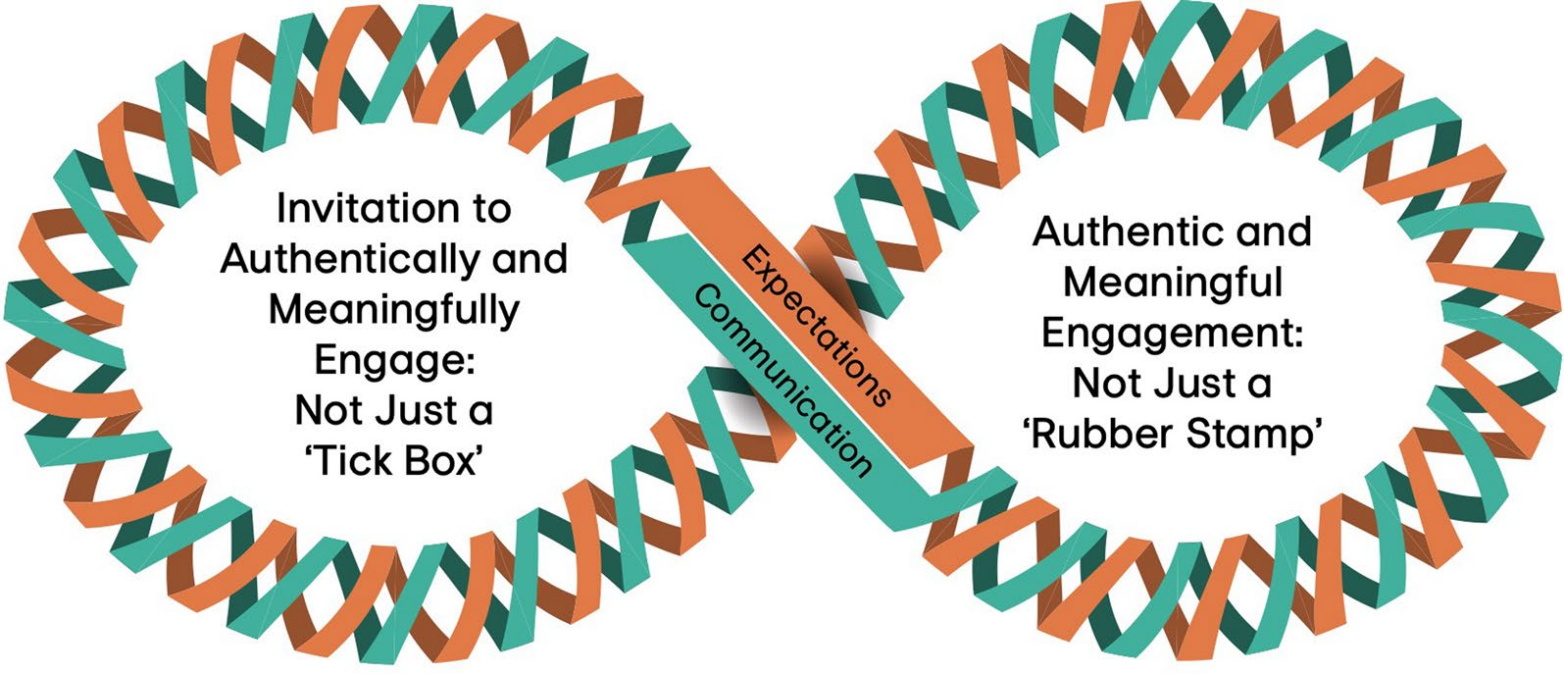
Communication and expectations as elements of authentic and meaningful engagement in research that link the themes

When interview participants were asked about whether the final simulations were reflective of their experiences in POR, two participants (1 researcher, 1 trainee) explained that communication and expectations were common threads that linked the two themes of authentic and meaningful engagement with respect to invitations to engage and how people are engaged during research. A lack of clear communication and mutually agreed upon expectations were described as “symptoms of underlying problem” (participant 5, research staff) that work to create conflicts or tension in research engagement. The relationship between these concepts was highly linked: “clear expectations and communication…relates to that issue of tokenism, and tokenism relates to this issue of barriers and equity…I think there are underlying themes around needing to give people both the skills and the tools to do this properly” (participant 5, research staff). Figure 2 illustrates an infinity loop depicting how expectations and communication continuously link the themes of invitation to engage beyond a “tick box” and to engage beyond a “rubber stamp” such that engagement in research is authentic and meaningful.

### Recommendations

We heard during the interviews that authentic and meaningful engagement can be achieved in a variety of ways including (1) setting mutual expectations; (2) building rapport; (3) offering tangible supports; (4) intentional collaboration; and (5) building in sufficient time into the project and creating space to collaborate. These recommendations are described in Table 1.

**Table 1:** Recommendations for achieving authentic and meaningful engagement in research

Recommendations	Description and examples
Set mutual expectations	Collaboratively outline and negotiate expectations about the project as a team (e.g., not the researcher alone) and discuss how researchers and families would like to work together from the beginning of a project. Revisit and reassess the expectations throughout the course of the project
	Do not assume motivations or experience. At the outset of a project explicitly discuss the patient or family partner’s skills, interests, and how they would like to meaningfully contribute to the project
	Research conducted with patient and family partners should be conducted in a way that is respectful and values all contributions
Build rapport	Build trust and continue to build and maintain social connections throughout the partnership
Offer tangible supports	Provide the research team with tangible supports (e.g., engagement tip sheet designed by patients and families themselves) and utilize pre-existing frameworks (e.g., around compensation or honoraria) to support authentic and meaningful engagement
Intentional communication	Thoughtful, open, and ongoing communication, as well as curiosity, can facilitate and support meaningful conversations between team members (e.g., checking in or providing updates about the project, even if there is not anything for the partner to do at that time)
Build in time and share space	Time is needed to build partnerships (e.g., building in opportunities such as additional time for teams to get to know each other and have informal discussions), while space (e.g., during discussions) is needed to give partners the opportunity to collaborate

## Discussion

Training that is codesigned with multiple stakeholders from different backgrounds has the potential to build learners’ capacity on a specific topic or subject by mimicking real-world problems and trying solutions in real time [33, 54]. We developed four simulation training videos with representatives of stakeholder groups who had experience partnering on research teams. Despite a growing movement to include patients and families as partners on research teams, our findings suggest that invitations to engage do not automatically lead to collaborative work that is authentic and meaningful. Participants described that engagement invitations often do not include individuals who are marginalized due to race or economic status. This is not a new issue in the patient engagement literature and others have written on the lack of diversity in POR [16, 55, 56]. Parking and other financial arrangements, while sometimes offered, may not be enough to off-set costs of engagement (e.g., childcare, transportation, language interpretation), thereby limiting who is included in the research process. Moreover, these items may not be routinely accounted for in research budgets, and some partners may have additional needs that go beyond these more common barriers to engagement.

Social and financial disparities (among others) often afford some more opportunities to collaborate in health research, since some groups may have different needs than those who can easily collaborate. If not invited to engage in this process, the needs of those excluded are unlikely to be fully represented in the research. In setting up the research project, Chauhan et al. suggest that liaising with community support groups and peer-led community groups prior to the research can build trust and help identify potential partners as a way to increase the diversity of partnership invitations [56]. This is consistent with the strategies to engage hard-to-reach populations in childhood disability described by Gonzalez et al. [16]. Additionally, Gonzalez et al. suggest other potential recruitment strategies, including parent support groups, having gatekeepers and clinicians identify potential partners, and having a partner of the same background as the target participants contact families within a familiar organization to the research team [16].

Clear communication and mutual expectations are pivotal to the engagement process and this is consistent with Hughes and Duffy whose concept analysis suggests that, for engagement to be meaningful, certain conditions and outcomes must exist, including identifying the goals and purpose of engagement, and sharing power [18]. Clearly outlining the roles and responsibilities that patient partners take on in a project is frequently cited as an important factor for meaningful engagement in reviews on patient partnerships [5, 8] and a key facilitator in setting up the research partnership [57]. Expectations, motivations, and needs of patient and family partners need to be addressed ideally during the initial planning phase when designing the research study, for example, by asking the reflective questions from the point of view of a patient or family partner (e.g., Why would I want to be part of this research study? What do I feel I can contribute? How do I hope my contribution will lead to real, positive change?). For families and researchers just beginning the partnership process, frameworks and tools can be helpful to identify what level of engagement might be appropriate given the goals of the project [21, 58, 59] and also, as participants in our study suggest, their knowledge, skills, and interests, such that roles and responsibilities are not decided solely on their lived experience. Having conversations at the outset of the project about the stories that motivates patients and families to participate and how and when they want to be involved in the research is thus critical to understanding how and when partners want to be involved. The research team should critically reflect on what they can do to ensure that all partners feel heard, and that they can contribute in ways that are meaningful to them.

Participants in this study described a need to understand how much their contributions are valued because people with lived experience are often not asked to share in all the ways they could contribute. Meaningful contributions are not just about putting a name or a ‘rubber stamp’ on a project—they were described as an understanding of those unique skills and interests that partners bring to a project and mutually deciding on ways to integrate that into the project. Anecdotally, our findings align with the experiences described by Curran et al. and the ‘lessons learned’ in their time partnering with parents to advance child health [60]. Curran et al. caution that “instances of tokenism in this sense could create a greater divide between researchers and the public and lead to less trust in the research community as a whole” (p. 48). Conceptually, what we see as meaningful engagement is perhaps similar to what Hahn et al., refer to as ‘genuine engagement’, which they contrast with ‘token engagement’ [17]. While we are unclear if tokenism is just the opposite of meaningful engagement, Hahn et al. suggest that “real engagement—to be involved in shared dialog and responsibilities, to build strong and lasting relationships between researchers and community/patient partners and to develop a research relationship that encourages this type of partnership” (p. 295) helps to move genuine engagement forward.

There are many risks if researchers and patient and family partners are not on the same page and if engagement is not genuine. They may experience strained relationships, feelings of disappointment or frustration, conflicts, and a potential discontinuation of the partnership [61]. Researchers may also be concerned about project delays or an inability to complete all project steps as planned [62]. While granting agencies are increasingly requiring patients and families to be involved as partners on research projects to gain funding, there is an associated risk of harm to the partner if there is no expectation or guidelines for family engagement during the planning and design stage of a project. Patient and family engagement needs to be part of these early phases of the research, and not just the execution phase, to help ensure authenticity. Hands-on training is seen as an essential way to build the fundamental skills required to authentically and meaningfully engage but few opportunities exist which allow researchers and patient and family partners to learn together in a safe and supportive environment (i.e., mutual respect, lack of hierarchy). Integrating simulation exercises, such as the videos described in this article, into existing training programs might be a valuable way to expand and reinforce the knowledge and skills these courses aim to build.

## Strengths and limitations

A range of perspectives were included in the design and conduct of this study. Two study team members were family partners, and one patient partner joined the study team after data collection to support analysis and publication. Not all codesign groups participated in the research, limiting the number of observation and interview participants and the transferability of study findings. Although our focus was on childhood disability research, we focused on youth with disabilities and their families and did not directly include the perspectives of children with disabilities. There is also a risk of bias as participants had to have experience in POR to participate in this work and were likely committed to the topic to choose to participate in the study.

## Reflections on patient engagement in this research study

This research was completed by a diverse team that was engaged throughout the research process. Partners shared that they had space to express challenges and share stories about working collaboratively in research. As a team, however, we found that it can be difficult to schedule team meetings and research activities that were a good fit for everyone. Although family partners want to be involved in the process, they may struggle to find time given other competing priorities (e.g., work, parenting, etc.). Challenges related to the simulation codesign included a lack of role clarity (e.g., “participant” vs. “partner”) and understanding of the codesign process. Logistics, such as timing of the simulation  codesign event, coordinating multiple codesign sessions, and integrating standardized patient actors into the codesign process can be difficult. We learned to be responsive to unexpected changes and to ensure that everyone is starting at the same place. The COVID-19 pandemic challenged us to complete the interviews online and record one simulation via Zoom (instead of in-person). Due to these circumstantial context challenges, we found it especially important to be transparent about roles and expectations and provide timelines for tasks, while being understanding of a challenging and ever-changing context impacted by a global pandemic.

## Future directions

As a team, we plan to continue exploring how simulation videos can be used to improve knowledge and attitudes about authentic and meaningful partnership in research and improve learners’ ability to engage in patient-oriented research. For example, the simulation videos developed and outlined in this article may act as a helpful training tool that promotes authentic and meaningful engagement in research. A necessary next step is the evaluation and validation of the simulation videos as a training tool to build knowledge and skills in patient and family engagement in research. There is a clear need for additional supports about how to engage in a meaningful and authentic way, particularly with children and youth with disabilities, as they were less represented in this research. Exploring the role of supports/caregivers in research and the strategies to facilitate engagement with young people with diverse communication or support needs should be a priority for more inclusive research. Given that there were a limited number of participants involved in this project and they were all highly committed to meaningful engagement in research, future work should be conducted with youth, caregivers and researchers who were not involved in the development of the simulations and who may have less experience with authentic and meaningfully engaging in research. Participants in this research suggest that there is a need for greater time to build partnerships and this is consistent with the broader patient engagement literature [17, 57]. Despite having knowledge of POR best practices, barriers to patient and family engagement such as lack of time and sufficient resources [63] may further constrain researchers from engaging with partners in an authentic and meaningful way. Therefore, we recommend (1) evaluation of the simulations as a training tool; (2) implementation of training to support authentic and meaningful engagement in research in which researchers and patient and family partners learn together; and (3) acknowledgement of the structural barriers to engaging in authentic and meaningful POR (e.g., time and resources) with advocacy and leadership that aim to improve the structural conditions for engaging patients and their families.

## Conclusion

Authentic and meaningful engagement is important for patients and families to feel that they are valued beyond a ‘tick box’ and that their contributions are more than a ‘rubber stamp’. However, opportunities for engagement do not always align with the skills and interests that patient and family partners bring onto research teams. We suggest that clear communication and mutual expectations are important for engagement to happen and that these need to be established early in the research process and then threaded throughout. In this article, we have described how we codesigned a novel approach to building researcher, patient, and family partner capacity to engage in meaningful ways on research teams. While some training exists, simulation videos that facilitate co-learning offer a unique way to build the knowledge and skill of meaningful engagement that could complement existing training opportunities. Recommendations for authentic and meaningful engagement include setting mutual expectations, building rapport, providing tangible supports, clear thoughtful communication, and building in time and space to work together. We encourage research teams to continue to explore authentic and meaningful engagement particularly for those who may face additional barriers to engagement.

## Supplementary Information

**Additional file 1.** GRIPP2 long form.

**Additional file 2.** List of simulation topics.

## Data Availability

Not applicable.

## Abbreviations

FER: Family engagement in research partners in research
PaCER: Patient and community engagement research
PiR: Partners in research
POR: Patient oriented research
PORCCH: Patient oriented research curriculum in child health
SP: Standardized patients
