# Supplementary material for: Codesigning simulations and analyzing the process to ascertain principles of authentic and meaningful research engagement in childhood disability research
Source: Res Involv Engagem. 2022 Nov 9;8:60. doi: 10.1186/s40900-022-00398-y (PMC9645736; doi:10.1186/s40900-022-00398-y)
Supplement: Supplementary file 2 — Additional file 2. List of simulation topics. [file 40900_2022_398_MOESM2_ESM.docx]

**Additional File 2: List of Simulation Topics**

**Simulation topic 1:** During the planning phase of a research study, clients, families, and researchers may disagree about the research question or objectives.

**Simulation topic 2:** Setting expectations about roles and responsibilities with all team members (youth, parents, researchers) can be challenging when clients and family members have competing demands and are not being paid for their time.

**Simulation topic 3:** During research team discussions, clients and families naturally speak from their own experience. It can be challenging to provide the perspective of the broader client/family experience.

**Simulation topic 4:** Clients, family members and researchers may interpret the study results differently and it may be difficult to arrive at some common interpretation (s) of the data.

**Simulation topic 5:** Team members may disagree about who to share study results with and about how and when results should be shared.
